# Depression, Anxiety, and Daily Activity Among Adolescents Before and During the COVID-19 Pandemic: Cross-sectional Survey Study

**DOI:** 10.2196/30702

**Published:** 2021-12-02

**Authors:** Anna Jolliff, Qianqian Zhao, Jens Eickhoff, Megan Moreno

**Affiliations:** 1 Department of Pediatrics School of Medicine and Public Health University of Wisconsin - Madison Madison, WI United States

**Keywords:** COVID-19, pandemic, adolescent, depression, anxiety, socioeconomic status, survey, mental health

## Abstract

**Background:**

The COVID-19 pandemic has resulted in significant changes to adolescents’ daily lives and, potentially, to their mental health. The pandemic has also disproportionately affected historically marginalized and at-risk communities, including people of color, socioeconomically disadvantaged people, people identifying as female, and youth.

**Objective:**

This study aimed to understand differences in depression and anxiety among 2 groups of adolescents in the United States before and during the COVID-19 pandemic, and to examine demographic and daily activity variables associated with depression and anxiety.

**Methods:**

Online surveys were distributed in 2019 and 2020. Demographic questions were asked at the time of enrollment, and included participants’ age, gender, race and ethnicity, and socioeconomic status (SES). The 8-item Patient Health Questionnaire was used to assess symptoms of depression, and the 7-item Generalized Anxiety Disorder scale was used to assess symptoms of anxiety. A total of 4 pandemic-specific daily activity questions were asked only of the pandemic group. Analyses of covariance compared depression and anxiety between prepandemic and pandemic groups. Demographic and lifestyle variables were included as covariates.

**Results:**

The sample comprised a total of 234 adolescents, with 100 participants in the prepandemic group and 134 participants in the pandemic group. Within the pandemic group, 94% (n=126) of adolescents reported being out of school due to the pandemic, and another 85.8% (n=115) and 57.1% (n=76) were prevented from extracurricular activities and exercise, respectively. Higher depression was seen in the pandemic group, with a least-squares adjusted mean of 7.62 (SD 1.36) compared to 6.28 (SD 1.42) in the prepandemic group, although the difference was not significant (*P*=.08). There was no significant difference in anxiety scores between the 2 groups (least-squares adjusted means 5.52, SD 1.30 vs 5.01, SD 1.36; *P*=.48). Within the pandemic group, lower SES was predictive of anxiety, such that those in the pandemic group of lower SES were more anxious than their higher-SES peers (least-squares adjusted means 11.17, SD 2.34 vs 8.66, SD 2.16; *P*=.02). Within the pandemic group, being out of work or school and not partaking in extracurricular activities or exercise due to the pandemic were not associated with higher depression or anxiety scores.

**Conclusions:**

In this study, neither being in the pandemic group nor experiencing changes in daily activity due to the pandemic was associated with higher depression or anxiety. However, we found that adolescents from lower SES backgrounds experienced significantly more anxiety during the pandemic than their more privileged peers. Both instrumental and mental health interventions for low-income adolescents are imperative.

## Introduction

### Background

COVID-19 resulted in significant changes to adolescents’ daily lives [1]. This pandemic led to a prolonged period of statewide school closures [2,3], social isolation, unemployment [4], millions of infections, and hundreds of thousands of deaths [5]. The pandemic also disproportionately affected historically marginalized and at-risk communities, including people of color, socioeconomically disadvantaged people, people identifying as female, and youth [6,7]. However, it is unclear whether the early days of the pandemic were associated with appreciable changes in adolescents’ mental health in the United States.

Early international research suggested that the COVID-19 pandemic was associated with elevated rates of depression and anxiety in adolescents [8]. Research performed in Spain and Italy found increases in anxiety, irritability, and restlessness among adolescents [9], while Chinese adolescents reported increases in depression and anxiety during the pandemic [8,10]. Research in Germany suggests that increases in depression, anxiety, and distress were especially pronounced among those with pre-existing mental illnesses [11]. However, much of the research on the pandemic thus far has focused on either adults or adolescents outside the United States [9]. Limited research on US adolescents during COVID-19 demonstrates increases in depression [12,13] and anxiety [13,14]. A systematic review published in 2021 [15] demonstrated that research on US adolescents was still in a nascent stage; out of 16 international studies on the psychological impacts of COVID-19 on children, only 2 focused on US participants [16,17]. This is an important gap, as the experience of American adolescents has been somewhat unique from that of adolescents in other countries; safety measures were implemented more slowly and with greater variability in the United States, and remained in effect longer than anticipated [18].

Adolescents did not all fare equally well during the COVID-19 pandemic. Existing research suggests that, during COVID-19, adolescent females scored higher in depression and anxiety than adolescent males [8,14,15,19-21]. However, because most of these studies are cross-sectional, it is not clear whether COVID-19 disproportionately affected female over male mental health, or whether the greater anxiety and depression among female adolescents simply reflects pre–COVID-19 differences [22]. Indeed, 1 longitudinal study found that although adolescent females reported greater depression than males during the pandemic, this was proportionate to their pre–COVID-19 differences in depression [20].

Existing research shows that people of lower socioeconomic status (SES) had worse mental health outcomes during COVID-19. Research shows that US adults with a lower income and less than $5000 in savings experienced greater depression during the pandemic [12]. A study of US adolescents showed that residing in a county with higher poverty levels during COVID-19 was associated with greater depression and anxiety [21]. These negative mental health consequences may be due to the disproportionately negative impact COVID-19 had on people of lower SES. This group experienced a higher incidence of COVID-19 [23] due to limited access to health information, lack of protective personal equipment, and reduced freedom to socially distance [24]. Once infected, lower SES also predicted invasive mechanical ventilation, intensive care unit admission, and mortality [23,25]. The reality of greater risk among those with lower SES may explain greater depression and anxiety in this group; however, it is unclear whether these differences would be perceptible in the earliest days of the pandemic in a sample of adolescents of lower SES.

Research shows that, during COVID-19, being a member of a historically marginalized racial or ethnic group was associated with suicidality [26] and depression [12]. The observed decline in mental health may have been due to the disproportionately negative impact of COVID-19 on racial and ethnic minority communities. Members of racial and ethnic minority groups are more likely to face structural inequities, including inadequate access to health care and overcrowded schools and neighborhoods, both of which contribute to COVID-19 transmission and mortality [25,27]. Although racial and ethnic minority status is closely related to SES in the United States [25], Kim and colleagues [28] attempted to disaggregate the effects of minority status from the effects of SES on COVID-19 outcomes. These authors found that racial minority status, independent of SES, predicted COVID-19 death rate [28]. This may be attributable to the intra- and intergenerational effects of systemic racism on African American individuals’ health and quality of health care.

Daily activities such as part-time work, school, extracurricular activities, and exercise are a source of social support for many adolescents. The sudden removal of these activities —and therefore, of social support—may have resulted in depression and anxiety among US adolescents. Research on Canadian adolescents has shown that having schoolwork to do during quarantine was associated with less depression, while exercise was associated with less loneliness [29]. Research on adolescent athletes shows that athletes involved in team sports experienced more depression during COVID-19 than their peers involved in individual sports, suggesting that the sudden absence of the social component may have contributed to poor mental health [21]. In 2020, Loades and colleagues [30] conducted a rapid systematic review of pre–COVID-19 studies in an attempt to predict the likely effects of social isolation and loneliness on the mental health of adolescents. Based on their sample of articles, these researchers predicted that social isolation due to COVID-19 would result in increased depression among previously healthy adolescents.

### This Study

In summary, more research is needed to determine whether American adolescents experienced greater anxiety and depression in the early days of the COVID-19 pandemic; whether this effect was more pronounced among female, socioeconomically disadvantaged, or racial and ethnic minority adolescents; and whether changes to daily activity were associated with negative mental health. This study contrasts the mental health of US adolescents surveyed prior to the pandemic with that of adolescents surveyed 2 weeks after many states had enacted statewide school closures [2]. Our hypotheses were as follows:

1. Participants surveyed during the pandemic will report greater depression and anxiety than participants surveyed prior to the pandemic.
2. Adolescents surveyed during the pandemic who identify as (A) female, (B) having lower SES, or (C) a racial or ethnic minority will report greater depression and anxiety than their peers surveyed during the pandemic.
3. Adolescents who were prevented from attending (A) work, (B) school, (C) extracurricular activities, or (D) exercise due to the pandemic will report greater depression and anxiety than their peers who were not prevented from carrying out these activities.

It is vital to test these hypotheses as a key step to understanding and designing interventions for adolescents experiencing negative mental health outcomes during and in the aftermath of the COVID-19 pandemic.

## Methods

### Overview

This 2-group cross-sectional study was a subproject of a larger study. All surveys were taken online. The first group of participants completed the surveys between October 2019 and February 2020, while the second group of responses were received from March 31 to April 3, 2020. This study was approved by the (blinded for review) Institutional Review Board.

### Recruitment

#### Participants

Eligible participants were aged 13-17 years, lived in the United States, and spoke English. The aims of the larger project included evaluating different recruitment approaches for participation in ecological momentary assessment research. Thus, potential subjects for this subproject were recruited using 2 recruitment methods, including Qualtrics and Facebook advertisements. The prepandemic group included participants recruited via Qualtrics and Facebook, while the pandemic group included participants recruited via Facebook only.

#### Facebook Recruitment

Participants were recruited via paid advertisements posted on Facebook. Advertisements were targeted at the parents of teenagers aged 13-17 years. Parents who clicked on the advertisement were invited to read the informed consent document. Those who provided consent were invited to pass the device to their child, who would then provide assent and complete the eligibility screening. Parents whose children were not with them to provide assent were prompted to enter their child’s phone number; the child would then receive a link to read the assent document and complete the eligibility screening.

#### Qualtrics Recruitment

Qualtrics is a service that partners with survey research companies to recruit research participants meeting specified inclusion criteria [31]. Qualtrics samples have been shown to be politically and demographically representative of the US population and can replicate known population-level effects [32,33]. Qualtrics sends survey invitations to eligible participants, and compensates participants with rewards, including gift cards, retail points, and airline miles. In this study, Qualtrics sent invitations to the parents of teenagers aged 13-17 years. Only parents who indicated that their children were currently with them and available to provide assent were allowed to complete the consent document. After providing consent, parents were prompted to pass the device to their child so that their child could provide assent and complete the eligibility screening.

### Survey Measures

#### Demographic Information

Demographic questions were asked at the time of enrollment, and included participants’ age, gender, race and ethnicity, and SES. For age, respondents could select a number between 13 and 17. For gender, response options were “female,” “male,” “nonbinary gender,” “female to male transgender, “male to female transgender,” and “other,” with the option to write in a response. For race and ethnicity, participants could select 1 or more of the following options: “White,” “Black/African American, “Asian or Pacific Islander,” “American Indian or Alaska Native, “Hispanic/Latino,” and “other,” with the option to write in a response. To assess SES, participants were asked whether they or their sibling were on free or reduced lunch, and response options were “yes,” “no,” and “I don’t know.”

#### Mental Health Outcomes

##### Depression

The 8-item Patient Health Questionnaire (PHQ-8) was used to assess symptoms of depression [34]. Participants were asked to rate the extent to which they had experienced certain symptoms, from “not at all” to “nearly every day.” Example symptoms include “poor appetite or overeating,” “feeling tired or having little energy,” and “feeling down, depressed, or hopeless.” Cutoff scores of 5, 10, and 15 were used to indicate mild, moderate, and severe depression, respectively. Internal consistency for the PHQ-8 is excellent (Cronbach α=.89) [35,36].

##### Anxiety

The 7-item Generalized Anxiety Disorder (GAD-7) scale was used to assess symptoms of anxiety [37]. Similar to the PHQ-8, participants were asked to rate the extent to which they had experienced certain symptoms, from “not at all” to “nearly every day.” Example symptoms include “not being able to stop or control worrying” and “feeling afraid as if something awful might happen.” As with the PHQ-8, cutoff scores of 5, 10, and 15 were used to indicate mild, moderate, and severe anxiety, respectively [37]. Internal consistency for the GAD-7 is excellent (Cronbach α=.92) [37].

##### Changes in Daily Activity Due to the Pandemic

A total of 4 pandemic-specific daily activity questions were asked only of the pandemic group. Participants were asked to indicate whether, at present, they were prevented from (1) work, (2) school, (3) extracurricular activities, or (4) exercise due to the pandemic. These were categorical variables, and for each question participants could select 1 of 4 responses: “yes,” “no,” “does not apply,” or “other,” with the option to write in a response.

### Procedure

All participants received a link by SMS text messaging to complete the survey. Participants had 3 days after receiving the text message to complete the survey, and participant responses were retained if they completed more than 50% of it. Participants recruited through Qualtrics were compensated through Qualtrics, and those recruited through Facebook were compensated for participation with a check sent to their mailing address.

### Analysis

#### Demographic Variables

Descriptive statistics were used to summarize participants’ age, race and ethnicity, and gender, as well as the percentage of participants who were out of work, school, extracurricular activities, or exercise due to the pandemic. Age was treated as a continuous variable. Given the small number of participants endorsing certain races and ethnicities (eg, Hispanic/Latino, Asian, Pacific Islander), respondents were categorized as non-Hispanic White, non-Hispanic Black, multirace, or other. Due to the small number of participants endorsing genders other than male or female, respondents were categorized as “male,” “female,” or “other.” Participants who indicated that they did not know if they or their siblings were on free or reduced lunch were categorized as not being on free or reduced lunch. Most participants reported being out of school due to the pandemic; thus, the answers “no,” “does not apply,” and “other” were combined in the multivariate analyses. To evaluate demographic differences between the prepandemic and pandemic groups, the Fisher exact test was used for categorical variables, a 2-sample *t* test was used for normally distributed continuous variables, and a Wilcoxon rank-sum nonparametric test was used for nonnormally distributed continuous variables.

#### Main Analyses

Statistical analyses were conducted using SAS software, version 9.4 (SAS Institute Inc) [38]. All reported *P* values were 2-sided, and *P*<.05 was used to define statistical significance. Hypothesis 1 predicted that being in the pandemic group would be associated with significantly higher depression and anxiety scores compared to the prepandemic group. Analysis of covariance (ANCOVA) was used to test this hypothesis. Anxiety and depression were entered as continuous dependent variables, rather than categorical ones, in order to maximize sensitivity. Group was entered as an independent variable, and demographic variables (age, race, gender, SES) were entered as covariates.

Hypothesis 2 stated that, within the pandemic group, participants identifying as female, as having a lower SES, or as a racial or ethnic minority would be associated with higher depression and anxiety scores. Hypothesis 3 stated that, within the pandemic group, being out of work, school, extracurricular activities, or exercise due to the pandemic would be associated with higher depression and anxiety scores. ANCOVA was again used to test these hypotheses. Anxiety and depression were entered as continuous dependent variables. Work, school, extracurricular activities, exercise, and demographic variables were entered as covariates.

We conducted a sensitivity analysis by excluding all Qualtrics respondents and rerunning the analyses. Furthermore, we evaluated interaction effects between the groups (pre–COVID-19 or COVID-19) and each demographic variable (age, gender, race and ethnicity, and SES) to examine whether further subgroup analysis by demographic characteristics was required.

## Results

### Participants

There were 100 participants in the prepandemic group, and 134 participants in the pandemic group, for a combined total of 234 participants. Demographic differences between the 2 groups are recorded in Table 1. The samples showed a similar distribution of gender, although statistically significant differences were noted in race, age, and the number of participants on free or reduced lunch. Within the prepandemic group, the mean PHQ-8 and GAD-7 scores were 5.27 (SD 5.06) and 4.67 (SD 5.22), respectively. In the pandemic group, the mean PHQ-8 and GAD-7 scores were 6.81 (SD 5.67) and 5.37 (SD 5.18), respectively. Regarding changes in daily activity during the pandemic, 94% (126/134) of participants reported that they were prevented from attending school during the pandemic, and 85.8% (115/134) were prevented from extracurricular activities. The percentage of participants out of work, school, extracurricular activities, or exercise due to the pandemic is shown in Table 2.

**Table 1:** Demographic information for prepandemic and pandemic samples (N=234).

Characteristic			Prepandemic (n=100)		Pandemic (n=134)		*P* value	
Age (years), mean (SD)			14.7 (1.3)		15.2 (1.4)		.01^a^	
**Gender, n (%)**								.49^b^
	Female	66 (66)		92 (68.7)				
	Male	34 (34)		40 (29.9)				
	Other	0 (0)		2 (0.01)				
**Race, n (%)**								<.001^b^
	White, non-Hispanic	56 (56)		103 (76.9)				
	Black, non-Hispanic	18 (18)		8 (6)				
	Multirace	11 (11)		12 (9)				
	Other	15 (15)		11 (8.2)				
**Qualified for free or reduced lunch, n (%)**								<.001^b^
	Yes	51 (51)		41 (30.6)				
	No/don’t know	49 (49)		93 (69.4)				

^a^*P* value from 2-sample *t* test or Wilcoxon nonparametric test.

^b^*P* value from the Fisher exact test.

**Table 2:** Participants out of work, school, extracurricular activities, or exercise due to the COVID-19 pandemic 2 weeks after nationwide school closures (n=134).

Activity			Participants
**Work, n (%)**			
	Yes	31 (23.1)	
	No	5 (3.7)	
	Does not apply	98 (73.1)	
	Other	0 (0)	
**School, n (%)**			
	Yes	126 (94.0)	
	No	4 (3.0)	
	Does not apply	2 (1.5)	
	Other	2 (1.5)	
**Extracurricular activities, n (%)**			
	Yes	115 (85.8)	
	No	4 (3.0)	
	Does not apply	15 (11.2)	
	Other	0 (0)	
**Exercise, n (%)**			
	Yes	76 (57.1)	
	No	28 (21.1)	
	Does not apply	20 (15.0)	
	Other	9 (6.8)	

### Hypothesis Testing

Hypothesis 1 stated that being in the pandemic group would be associated with significantly higher depression and anxiety scores. In the adjusted model for depression, the pandemic group exhibited higher depression, with a least-squares adjusted mean of 7.62 (SD 1.36) compared to 6.28 (SD 1.42) in the prepandemic group; however, this difference was statistically insignificant (*P*=.08). Across both groups, female participants had greater depression scores than males (least-squares adjusted means 6.45, SD 0.55 vs 4.70, SD 0.69; *P*=.02). For anxiety, there was no significant difference in anxiety scores between the pandemic and prepandemic groups (least-squares adjusted means 5.52, SD 1.30 vs 5.01, SD 1.36; *P*=.48). Again, across both groups, females reported greater anxiety than males (least-squares adjusted means 5.59, SD 0.53 vs 3.58, SD 0.66; *P*=.01). No other demographic variables were significant predictors of depression or anxiety.

Hypothesis 2 stated that, within the pandemic group, identifying as female, as having a lower SES, or as a racial or ethnic minority would be associated with greater depression and anxiety. In the adjusted model for depression, none of these variables were significant predictors of depression. In the adjusted model for anxiety, being eligible for free or reduced lunch was associated with greater anxiety, such that those in the pandemic group who qualified for free or reduced lunch were more anxious than their peers who did not (least-squares adjusted means 11.17, SD 2.34 vs 8.66, SD 2.16; *P*=.02).

Hypothesis 3 stated that, within the pandemic group, being out of work, school, extracurricular activities, or exercise due to the pandemic would be associated with higher depression and anxiety scores. No significant associations were detected.

When we conducted a sensitivity analysis by excluding the Qualtrics participants and rerunning the analyses, the results did not change. We also did not detect any significant interaction effect between the groups (pre–COVID-19 or COVID-19) and the demographic characteristics. Therefore, no additional subgroup analyses by demographic characteristics were conducted.

## Discussion

### Principal Findings

This study examined differences in mental health between adolescents surveyed prior to the COVID-19 pandemic and those surveyed approximately 2 weeks after many states had enacted statewide school closures. Contrary to our hypothesis, adolescents in the pandemic group did not score significantly higher in depression and anxiety after adjusting for covariates. Within the pandemic group, lower SES was associated with higher anxiety. The majority of adolescents were prevented from attending school or participating in extracurricular activities and exercise due to the pandemic, and these changes in daily activity were not associated with depression or anxiety. These findings have important implications for future research on the adolescent experience of the COVID-19 pandemic.

First, we expected to find higher depression and anxiety scores in the pandemic group. This potential finding would have been consistent with existing international literature on COVID-19, which has shown that adolescents and young adults experienced elevated depression and anxiety due to the pandemic [8-10]. In our study, after controlling for the effects of demographic variables, being in the pandemic group was not associated with higher depression or anxiety. It may be that, after only 2 weeks in quarantine, American adolescents did not experience the pandemic as depressing or anxiety-inducing. They may have thought that the restrictions would quickly be lifted or, like many, may not have foreseen the gravity of the virus. Research completed several months into quarantine could yield different results. However, in both the prepandemic and pandemic groups, being female was associated with greater PHQ-8 and GAD-7 scores. This is consistent with existing literature, showing that adolescent females are more at risk for depression and anxiety [22].

In the group of participants surveyed during the pandemic, neither gender nor race was associated with depression or anxiety, which is inconsistent with previously cited research on gender [8,20,21] and race [25-28] during COVID-19. However, SES was associated with anxiety, such that participants of lower SES reported greater anxiety. This is consistent with existing research, which showed that SES was a risk factor for worse mental health outcomes during the pandemic [12,21,39]. It may be that COVID-19 magnified disparities that were not statistically significant in the prepandemic sample. Families of lower SES encountered more COVID-19 infections and fatalities, which may directly challenge the mental health of both infected people and their loved ones [23,25,40]. Underresourced adolescents, in particular, often rely on school for affordable food and school-based health care [41], and may have experienced greater anxiety when those resources were no longer available. In the wake of COVID-19, interventions should focus on adolescents of lower SES as their well-being is uniquely at risk due to the pandemic.

Our results showed that 2 weeks after many school closures, 94% of participants were prevented from going to school due to the pandemic. The majority of participants were prevented from partaking in extracurricular activities or exercise due to the pandemic, perhaps because these opportunities are tied to school for many adolescents. However, these disruptions to daily life within the pandemic group were not associated with higher depression and anxiety scores. This may have occurred for multiple reasons. First, only 2 weeks into quarantine, the absence of daily activities may have felt like a welcome reprieve, especially for adolescents with social anxiety [42], as well as students who experience bullying [43] or discrimination [44,45] in the context of their daily activities. Second, adolescents in this study may have experienced more opportunities for prosocial behavior as a result of the pandemic, such as helping, caring for, and comforting friends and family members; prosocial behavior is associated with positive adjustment [46]. Last, adolescents have a demonstrated ability to use technology to navigate developmental and mental health challenges; in our study, adolescents may have found entertainment, support, and distraction through digital interaction, such that any effects of COVID-19 on mental health were mitigated [47].

### Limitations

This study was not without limitations. First, the prepandemic and pandemic groups were significantly different in terms of age, race and ethnicity, and qualifying for free or reduced lunch. Additionally, prepandemic data were collected in the fall and winter months, while pandemic data was collected in the spring. This may have obscured differences caused by the pandemic. However, with the exception of season, ANCOVA should have controlled for all group differences. Second, the majority of participants did not work, making it impossible to ascertain the effects of the pandemic on adolescents who lost employment. Third, there may be constructs better suited than “anxiety” and “depression” for understanding adolescents’ experience of the pandemic. Negative experiences such as fear, boredom, or confusion may be more appropriate, as might positive experiences such as feeling less social or academic pressure, or feeling more supportive and supported online and offline. Further, the 4 pandemic-specific daily activity questions were not based on any validated scales, so these results should be interpreted with caution. Last, because data were collected in early April, findings may not be generalizable to the later periods of the pandemic.

### Conclusion

This research suggested that, at least in the early days of the pandemic, adolescents had not developed significantly greater anxiety or depression at the population level. Further, 2 weeks after many school closures, reductions in daily activities were not associated with anxiety and depression. However, we found that adolescents from lower socioeconomic backgrounds experienced significantly more anxiety during the pandemic than their peers of higher SES. Both instrumental and mental health interventions for adolescents of lower SES are imperative. More research is needed to understand the trajectory of adolescents’ mental health experiences during the COVID-19 pandemic, as well as the long-term impact of the pandemic on adolescent mental health.

## Abbreviations

ANCOVA: analysis of covariance
GAD-7: 7-item Generalized Anxiety Disorder
PHQ-8: 8-item Patient Health Questionnaire
SES: socioeconomic status
